# Editorial: Non-cadherin based cell adhesion in tissue remodeling

**DOI:** 10.3389/fcell.2024.1408075

**Published:** 2024-04-11

**Authors:** Lara Carvalho, Dan T. Bergstralh, Inês Cristo, Floris Bosveld

**Affiliations:** ^1^ iNOVA4Health, NOVA Medical School, Universidade Nova de Lisboa, Lisbon, Portugal; ^2^ Division of Biological Sciences, University of Missouri, Columbia, MO, United States; ^3^ Cardiovascular Disease and Regeneration Unit, Centro Cardiovascular da Faculdade de Medicina (CCUL@RISE), Universidade de Lisboa, Lisbon, Portugal; ^4^ Genetics and Developmental Biology Unit, Institut Curie, Université PSL, Sorbonne Université, CNRS UMR3215, INSERM U934, Paris, France

**Keywords:** cell junctions, atypical cadherins, desmosomes, integrin-based adhesions, occluding junctions, focal adhesion, cell adhension, model organisms

Cell junctions play central roles in regulating cell-cell interactions, tissue architecture and collective cellular behaviors during tissue and organ development and disease progression. As dynamic structures, junctions can be more or less stable and interact with different cytoskeletal networks and signaling pathways depending on their molecular composition and cellular context. While much research has focused on Adherens Junctions due to their critical roles in cell-cell adhesion and mechanotransduction, other types of junctional complexes, such as occluding junctions and desmosomes, have received less attention.

This Research Topic brings together a collection of review, methods, and research articles that offer new insights into the roles of different types of cell-cell contacts and cell adhesions, from occluding junctions and desmosomes to atypical cadherin adhesions and integrin-based contacts, during tissue remodeling. Importantly, this Research Topic includes studies covering diverse tissue contexts and cell types, ranging from cell migration and tissue patterning during development to the pathogenesis of genetic diseases. This diversity of research is made possible by taking advantage of established model organisms—including flies, frogs, and mice—and emerging systems, such as echinoderms. Below is a brief overview of the articles included in this Research Topic:

Two articles delve into the role of atypical cadherins in tissue polarity, patterning, and growth. Gridnev and Misra provide a review of the recent literature on the role the Dachsous-Fat signaling pathway in tissue planar cell polarity and growth and how these two facets of morphogenesis are coordinated. They discuss the key components of this pathway in *Drosophila*, namely, the atypical cadherins Dachsous and Fat, and their interplay with other signaling pathways, such as the Hippo pathway. Furthermore, this review summarizes the limited yet significant recent findings regarding Dachsous and Fat in mammalian systems. Regarding also atypical cadherins in mammals, Basta et al. investigate the role of two additional proteins, Celsr1 and Celsr2, in establishing planar cell polarity in the mammalian skin. Their study reveals that Celsr1 plays a more critical role than Celsr2 in the establishment of hair follicle polarization in the mouse epidermis. By comparing the phenotypes of single and double mutant mice for Celsr1 and Celsr2, the authors propose that these two related proteins exhibit distinct adhesive properties, which may underlie their different functions in development. Specifically, Celsr1 appears to be more crucial in static tissues like the skin, while Celsr2 may be more relevant in more dynamic processes, such as neuronal migration.

Two additional articles focus on the role of occluding junctions and integrin-based adhesions in *Drosophila* dorsal closure movements, a well-established model for studying epithelial tissue morphogenesis. During dorsal closure, epithelial cells undergo coordinated and dramatic cell shape changes and movements to seal a dorsal gap occupied by an extraembryonic tissue called the amnioserosa. De et al. demonstrate that core components of septate junctions, a type of occluding junction in invertebrate organisms thought to be the functional homologues of vertebrate tight junctions, are required for the final stages of dorsal closure. Mutations affecting septate junctions result in defects in cell shapes, leading to tissue tears and a halt in dorsal closure. The authors propose that septate junctions play a crucial role in maintaining cell adhesion between the epidermis and the amnioserosa, possibly through interactions with adherens junctions. Meanwhile, Karkali et al. explore the involvement of integrins in this process. They show that integrin receptors engage in a positive feedback loop with Jun kinase signaling to promote tissue integrity during dorsal closure movements.

Kho et al. reveal new insights about the regulation of focal adhesions during neural crest cell migration in frogs. Focal adhesions are adhesive points that link cells to the extracellular matrix, through integrin receptors and the F-actin cytoskeleton. In this study, the authors demonstrate that septin filaments play a crucial role in guiding both the speed and direction of neural crest cell migration by regulating focal adhesion and actin stress fiber stability and contractility. Whether septin filaments regulate focal adhesions in other types of migrating cells will be an interesting Research Topic of further investigations.

Barone and Lyons propose an evolutionary approach to the study of tissue morphogenesis. As central players in the development of virtually all multicellular organisms, the evolution of cell adhesion components is intimately connected with the evolution of multicellularity. Various types of cellular junctions found in vertebrates are also present in early-branching taxa, such as cnidarians. However, many gaps remain in the evolutionary tree. Studying the early developmental stages of echinoderms is starting to shed light on the evolutionary conservation of cell adhesion molecules and mechanisms. In their methods paper, Barone and Lyons describe a set of cutting-edge yet accessible methodologies for live imaging of echinoderm embryos that allow the visualization and analysis of cellular behaviors and tissue morphogenesis in a three-dimensional way, with subcellular resolution—an endeavor that can be challenging in more complex organisms.

Finally, a glimpse on the relevance of cell adhesion in the pathogenesis of cardiac diseases is provided by Shoykhet et al. These authors explore the impact of manipulating the metalloproteinase ADAM17 on cell adhesion in cardiomyocytes in mice. Their findings reveal that inhibiting ADAM17 prevents the cleavage of the protein Desmoglein 2, a crucial component of desmosomes, leading to its accumulation at cell-cell contacts and a consequent increase in cardiomyocyte cohesion. The authors suggest that targeting ADAM17 could serve as a therapeutic strategy to restore cardiomyocyte cohesion in the context of arrhythmogenic cardiomyopathy—a genetic disorder often linked to mutations in desmosome-related proteins. Moreover, this research may extend to other diseases characterized by desmosome dysregulation, such as pemphigus vulgaris, which manifests as blister formation in the skin and mucous membranes.

Altogether, this Research Topic provides an updated perspective on the range of cell adhesion mechanisms across various models and contexts. It highlights the need to further investigate the relevance of cellular junctions beyond Adherens Junctions in tissue remodeling throughout development and disease. We also anticipate this research will foster the development of new tools for analyzing tissue remodeling in a more comprehensive, three-dimensional manner.

